# A feasibility study of an exercise intervention to educate and promote health and well-being among medical students: the ‘MED-WELL’ programme

**DOI:** 10.1186/s12909-020-02097-2

**Published:** 2020-06-03

**Authors:** Aubree Worobetz, Petrus J. Retief, Sinead Loughran, Jane Walsh, Monica Casey, Peter Hayes, Enrique García Bengoechea, Andrew O’Regan, Catherine Woods, Dervla Kelly, Raymond O. Connor, Deirdre Mc Grath, Liam G. Glynn

**Affiliations:** 1 Graduate Entry Medical School, University of Limerick, Limerick, Ireland; 2 Department of Psychology, National University of Ireland, Galway, Ireland; 3 Health Research Institute, University of Limerick, Limerick, Ireland; 4 Department of Physical Education and Sport Sciences, Physical Activity for Health Research Cluster, University of Limerick, Limerick, Ireland

**Keywords:** Feasibility study, Medical education, Physical activity

## Abstract

**Background:**

Medical School programme workloads challenge the physical and mental health of students particularly in compressed graduate entry programmes. There is evidence that physical activity (PA) can improve holistic care and help maintain wellness among medical students. We tested the feasibility of introducing an exercise programme to the medical school curriculum which would educate and promote health and well-being among its students.

**Methods:**

This study was conducted in a single graduate entry medical school at the University of Limerick (UL). The ‘MED-WELL’ programme was a six-week programme of 1 hour-long weekly sessions, each involving a different type of PA (45 min). These sessions were prefaced by an interactive lecture about how to incorporate exercise theory into daily medical practice (15 min). The study was conducted in a single graduate entry medical school at UL and involved year one and year two graduate entry medical students. Three parameters were used to test feasibility: 1. Recruitment and retention of participants, 2. Acceptability of the programme and 3. Efficacy in terms of health and well-being. The latter was assessed by administering questionnaires pre and post the intervention. The questionnaires used the following validated measurement scales: EQ-VAS; WHO-5 Well-Being Index; 3-item Loneliness Scale; Social Support Measure 3-item scale. Free text boxes also encouraged participants to discuss the merits of the programme.

**Results:**

In total, 26% (74/286 students) participated in the programme. Of those who participated, 69 students (93%) attended one or more sessions of the programme and completed questionnaires at baseline and at follow-up. Significant improvements were seen in scores after the programme in the WHO-5 Well-Being Index which increased from 63.2 (95%CI: 48–78.4) to 67.5 (95%CI: 55.1–79.9); (*P* < 0.01), the sleep scale which increased from 3.1 (95%CI: 2.2–4.0) to 3.5 (95%CI: 2.5–4.5); (*P* < 0.001), and the loneliness scale which decreased from 4.1 (95%CI: 2.7–5.5) to 3.5 (95%CI: 2.5–4.5); (*P* < 0.005). Students level of PA during a typical week also increased from 3.7 (95%CI: 2.1–5.4) to 4.0 (95%CI, 3.5–4.5); (*P* < 0.05).

**Conclusion:**

This study has shown it is feasible to deliver this programme in a medical school’s curriculum. The programme seems to be of benefit and is acceptable to students. Well-designed randomised controlled trials are needed to measure outcomes, durability of effect, and cost effectiveness.

## Background

Medical school programmes challenge the mental health of students particularly those in compressed graduate entry programmes. A condensed medical degree of 4 years is accompanied by high workloads and demanding schedules. These academic pressures are often associated with increased anxiety and lower moods in otherwise healthy medical students, especially during examination periods [1]**.** Medical students have been shown to have high levels of suicidal ideation with a meta-analysis demonstrating a prevalence of suicidal ideation in medical students of 11.1% [2]. The same meta-analysis also found high levels of depression in medical students compared to the general population with a prevalence of depression or depressive symptoms in medical students of 27.2% [2]. A study from the United States of America specifically looked at medical students compared to other age-similar college degrees and found that medical student mental health deteriorates faster despite beginning their degrees with similar levels of mental health as the matched students [3]. While appropriate levels of stress can be conducive to improved academic performance, excess stress results in decreased cognitive functioning, which can have a negative impact on academic performance [1].

Psychosocial factors are significant predictors of performance in medical school [4]. Loneliness is a major influencing factor in an individual’s psychological health and has been proposed to have negative correlations with both social support and wellbeing [5]. In a group of British undergraduate students higher levels of loneliness predicted higher levels of anxiety, stress, and depression over time [6]. There is also clear evidence which suggests that medical students are already at a higher risk of developing these disorders when compared to their University peers [1, 2].

The Irish national PA guidelines recommend at least 30 min of moderate to vigorous PA 5 days per week, or 150 min over a week [7]. Individuals who meet these guidelines often benefit not only in their physical health, but mental health as well [8]. For students in particular exercise as a method of intervention has been shown to be beneficial to their overall wellbeing [9, 10]. Exercise is suggested to reduce stress, and aid sleep, which can improve overall academic performance [11] and cognitive functioning [1]. Conversely lower levels of exercise, specifically in medical students, have been related to higher levels of burnout [12]. One study of 279 medical students found over half of the participants were not meeting the recommended levels of PA with study-related activities being one of the main barriers to engaging in PA [13].

Medical schools worldwide vary greatly in their approach to the integration of exercise as a topic into medical school curricula. However this inclusion into the curricula has been slow and inconsistent. In some cases specific types of exercise have been integrated into the schedule of medical students such as group fitness classes or yoga [14, 15]*.* These interventions improved overall health of medical students however the interventions did not include an educational component on how to incorporate exercise theory into medical practice. Our aim is to involve students in using exercise as a tool to maintain their own health but also to educate them on exercise as medicine. In 2012, a United Kingdom survey on undergraduate medicine training estimated that only 56% of medical schools had incorporated some form of exercise medicine into their curricula [16]. Another study in the United States of America in 2013 found that the majority of medical education curricula did not offer any exercise education-related courses [17]. Concurrently, many future doctors indicate that they want to receive more teaching on PA through workshops on exercise prescription and through Problem-Based Learning (PBL) [18].

The aim of this study was to test the feasibility of an exercise intervention – the ‘MED-WELL’ programme – which would educate students about the benefits of and how to prescribe exercise as medicine, and also promote health and well-being among medical students themselves.

The specific objectives were to determine: 1) study feasibility, including: recruitment, retention, and assessment of outcome measures, and 2) intervention feasibility, including: intervention fidelity, efficacy, acceptability, and potential of medical schools to deliver the intervention.

This study will facilitate preparation for a phase II pilot randomised controlled trial of this intervention according to the Medical Research Council evaluation framework.

## Methods

### Study design

#### Feasibility study

##### Setting

This study was conducted in a single graduate entry medical school at the University of Limerick in the West of Ireland. This is the newest medical school in Ireland and was commissioned in response to the Buttimer report [19]. The medical school runs a 4 year graduate entry programme and graduated its first cohort of students in 2011, a class of just 32 students. It has now reached a steady state of graduating approximately 150 students per year.

An extensive consultation process was carried out with the student body, administration staff, and faculty to agree on the most suitable time for the ‘MED-WELL’ programme to take place within the medical school curriculum. Wednesday morning at 8:30 am was chosen as the most suitable time for the exercise intervention as there was minimal scheduling conflict with academic or extra-curricular commitments at that time.

### Participants

All year one and year two medical students of the 2018–2019 academic year at the graduate entry medical school, UL were eligible to participate in the ‘MED-WELL’ programme. Years one and two of the 4 year programme incorporate pre-clinical training through a PBL curriculum. Participation in the ‘MED-WELL’ programme was voluntary and not linked to assessment of the students in any way. It was not compulsory for participants to attend all sessions and if sessions were missed the participants were welcomed to return in subsequent sessions. Students were invited to participate via notification at the school (recruitment posters, student society promotion including Facebook and Twitter, and student email) 3 weeks in advance of the start date. Weekly email reminders were also sent to the students throughout the six-week intervention where they were encouraged to attend any sessions possible.

### Data collection

Before initiation of the programme informed signed consent was obtained. A pre-programme questionnaire was completed by each participant. Attendance was not compulsory but was recorded for each session, and a follow-up questionnaire was completed by each participant within 4 weeks of the final session of the ‘MED-WELL’ programme. Self-reported outcomes were evaluated before and after the ‘MED-WELL’ programme.

The pre-programme questionnaire examined the participant’s beliefs about exercise and exercise as medicine personally and professionally; current level of PA; current state of health and wellbeing; and measures of sleep pattern, concentration, and social support. It also asked subjective baseline questions about the participant’s PA such as their access to exercise resources, if they are a member of any sport or exercise-related clubs, societies or teams, if they use exercise or sleep tracking devices, or if they believe they have prior knowledge about the use of exercise as medicine.

After the 6 week intervention, a post-programme questionnaire was administered which examined the same parameters. The post-programme questionnaire also asked for an evaluation of the ‘MED-WELL’ programme and whether the participant would like to see it held every year.

Current level of PA was measured in both questionnaires via a two-part question which asked the participant to record their PA over the last 7 days and over a typical week. The participant was asked how many days they were physically active for at least 30 minutes at a moderate or vigorous intensity. Moderate intensity was defined as effort that makes the participant feel warmer, increases their heart and breathing rate, and may cause sweating but does not interfere with the ability to carry on a conversation. Vigorous intensity was defined as effort that increases heart and breathing rate, and causes deeper breathing and sweating.

The following validated measurement scales were also used in both questionnaires: EQ-5D-5 L VAS [20]; WHO-5 Well-Being Index [21]; modified version of the UCLA LS-8 scale [22]; Social Support Measure 3-item scale [23].

The EQ-5D-5 L VAS is a validated measurement of health status in subjects. Each participant in the study was asked to indicate their level of health at that moment, based on a 20 cm scale from 0 to 100; 0 representing “the worst health you can imagine”, and 100 representing “the best health you can imagine”.

The WHO-5 Well-Being Index is a self-reported measurement of current mental wellbeing and has been utilised in a variety of clinical settings. It consists of five statements related to mental wellbeing rated on a six-point Likert scale, where higher scores indicate better wellbeing. The WHO-5 Well-Being Index has also been proposed to be beneficial in highlighting mental health problems in college students, such as anxiety and depression [24].

A modified version of the UCLA LS-8 scale was used to represent the perception of loneliness [22]. Three questions from the original eight were used as they appropriately conveyed feelings of social isolation and loneliness in subjects. These questions are: “How often do you feel that you lack companionship?”, “How often do you feel left out?”, and “How often do you feel isolated from others?” A higher score conveyed an increased feeling of loneliness and social isolation.

The questionnaires also incorporated a 3-item Social Support Measure scale [23]. The questions used in the study encompassed physical presence of another individual during PA as well as perceived social support, whereby the subject had received external encouragement or felt supported while participating in PA.

A pilot of the questionnaires that were used in the study was completed before the study commenced. We piloted the questionnaires to ensure that they were acceptable and comprehensible to participants, and that our methods of administration were feasible and reliable.

### Intervention

The ‘MED-WELL’ programme intervention consisted of six weekly sessions lasting 1 hour each. At the beginning of each session, a health care professional spoke for 15 min to the participants on an exercise related topic (Table 1). The related slides for each pre-session talk were made available for the students on an online learning management system (Sulis) prior to each session. The remaining 45 min of each session consisted of PA adapted to different ability levels by a professional instructor with the type of PA varying each week (Table 1).

**Table 1:** Six-Week ‘MED-WELL’ programme schedule

Exercise Curriculum	Educational Curriculum	Educator
Week 1: Sports Yoga	Exercise as Medicine: PA for student wellness and patient use for exercise as a treatment	Academic General practitioner
Week 2: High Intensity Interval Training	Practical Applications: Prescribing PA for patients with chronic diseases: Case study of Diabetes	Academic General practitioner
Week 3: Body Pump	PA importance, regular health enhancing Dose Response Curve; Understanding people and PA stages of change (SOC-PA)	Academic in PA and health
Week 4: Pilates	Why not exercise? Overcoming resistance	Academic General practitioner
Week 5: Pilates - Respiratory Focus	Staying motivated for exercise	Academic Pharmacist
Week 6: Sports Yoga using drum-beat	Behavior Change: Relating behavior change wheel to PA promotion in practice	Research fellow

Feasibility of the intervention was measured by:

1. Examining the recruitment and retention of students (via sign-in). We considered 69 students acceptable as participation was voluntary and not linked to assessment of the students in any way,
2. Analysing the outcome measures showing the efficacy potential of the ‘MED-WELL’ programme (via pre- and post-programme questionnaire results). By analysing the outcome measures we could demonstrate that students’ health and wellbeing would benefit globally by participating, and
3. Questioning whether the programme was acceptable to medical students. This was assessed by a qualitative analysis of free text data from the post-programme questionnaire.

### Statistical analysis

A formal sample size for this feasibility study was not calculated [25]. Baseline data was summarised using suitable numerical summaries. The distribution of changes from baseline to follow up for each numeric outcome variable was tested for normality and paired samples t-tests were used for normally distributed changes. Mean changes with 95% confidence intervals (CI) are reported. A 5% level of significance was used for all tests. All statistical analyses were performed using the software package SPSS (version 24.0).

### Qualitative analysis

**Fig. 1:**
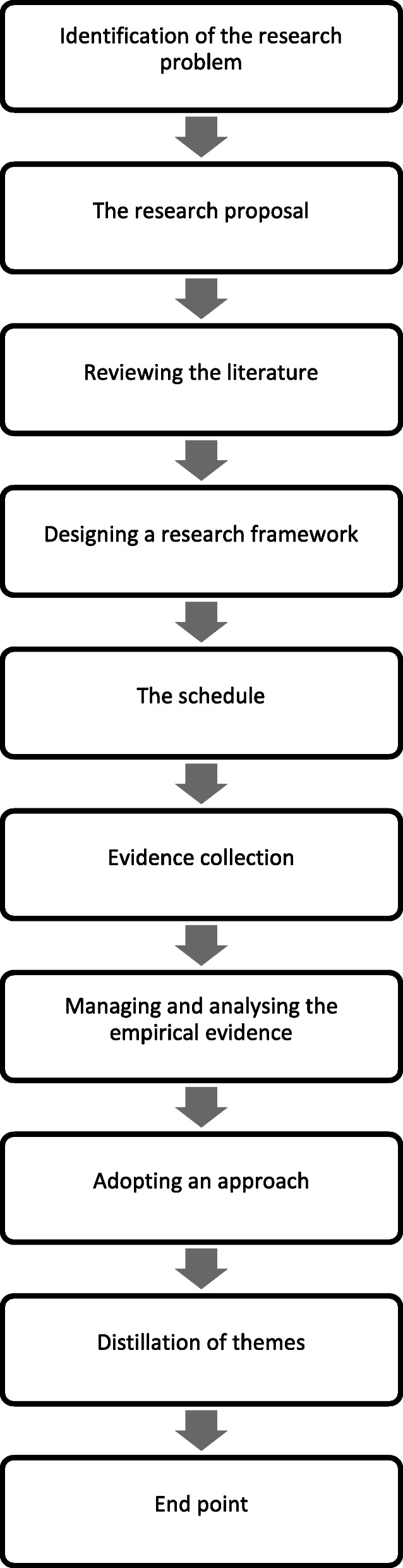
Summary of the physical audit trail

A physical audit trail was carried out which details the stages of the study and outlines key decisions in the research methodology [26]. The physical audit trail is summarized in Fig. 1 and is as follows:

#### Identification of the research problem

Medical school programme workloads challenge the mental health of students particularly in compressed graduate entry medical programmes. There is evidence that PA can improve holistic care and help maintain wellness among medical students. We would like to test the feasibility of introducing an exercise programme into the medical school curriculum which would educate and promote health and well-being among its students.

#### The research proposal

Based on the identified research problem a proposal was developed by students and faculty and submitted to the graduate entry medical school’s senior management team for approval. The proposal outlined the six-week ‘MED-WELL programme’ including the types of PA offered at each session alongside details of the educational component. The proposal also outlined where the sessions would take place, the duration of each session, any curricular space or assessment issues, and lastly how feasibility would be assessed.

#### Reviewing the literature

An in-depth review of the literature was undertaken. The review focused on current effects of PA on student wellbeing, as well as ways in which exercise or education on exercise as medicine is incorporated into other medical school curriculums.

#### Designing a research framework

The next step involved designing a research framework to support the collection of empirical evidence to assess the feasibility of the program. These parameters can be summarized as examining the recruitment and retention of students, analysing the outcome measures showing the efficacy potential of the ‘MED-WELL’ programme, and lastly questioning whether the programme was acceptable to medical students.

#### The schedule

The pre-programme questionnaire examined certain characteristics and beliefs of the participants which are fully described in Tables 2 and 3. At the end of the ‘MED-WELL’ programme a post-programme questionnaire was administered which examined similar parameters. The post-programme questionnaire also asked participants to comment about their experience in the ‘MED-WELL’ programme via free text boxes.

**Table 2:** Baseline characteristics of participants

Baseline Characteristic	Results	
Age	Mean 25 years; Range 21–41 years	
Gender	16 (22%) male	56 (78%) female
Medical School Year	28 (39%) Year 1	44 (61%) Year 2
Smoking	68 (94%) non-smoker; 1 (1%) smoker; 3 (4%) ex-smoker	
Do you have any medical conditions that prohibit your plan to exercise?	1 (1%) Yes	71 (99%) No
Do you have easy access to exercise resources (gym/teams/pitches)?	67 (93%) Yes	5 (7%) No
Are you a member of any clubs, societies or teams involved in sport/exercise?	40 (56%) Yes	32 (44%) No
Do you use any tracking devices to examine your levels of physical activity or sleep?	31 (43%) Yes	41 (57%) No
Has your level of exercise reduced since starting medical school?	42 (58%) Yes	30 (42%) No
Do you have any prior knowledge about the use of ‘Exercise as Medicine’ for patients with chronic illnesses?	33 (46%) Yes	39 (54%) No

**Table 3:** Mean difference in parameters between baseline and follow-up

Measurement	Baseline mean ± SD	Follow-up mean ± SD	*P* value
During the last 7 days, on how many days were you physically active at a moderate^a^ or vigorous^b^ intensity for a total of at least 30 min per day?	3.46 (± 1.8358)	3.70 (± 1.575)	0.178
On a typical or usual week, on how many days were you physically active at a moderate^a^ or vigorous^b^ intensity for a total of at least 30 min per day?	3.73 (± 1.6552)	4.00 (± 1.534)	0.021
On a scale of 1–4, how important do you perceive exercise as a treatment modality for many common chronic conditions (e.g. Diabetes, Osteoarthritis, COPD?)	3.65 (± 0.538)	3.81 (± 0.493)	0.062
On a scale of 1–4, how important do you perceive exercise as a modality to improve your personal health and well-being to improve your personal health and well-being and help prevention of chronic disease?	3.91 (± 0.284)	3.83 (± 0.419)	0.109
EQ-VAS	72.32 (± 13.404)	74.84 (± 12.532)	0.071
WHO-5 Well-Being Index	63.18 (± 15.197)	67.47 (±12.438)	0.015
Sleep Pattern	3.06 (± 0.879)	3.47 (± 0.985)	0.000
Concentration in Tutorials, Study and Lectures	3.43 (± 0.816)	3.60 (±0.736)	0.077
Loneliness Scale	4.09 (± 1.411)	3.64 (± 1.150)	0.003
Social Support Measure	14.67 (± 4.841)	14.90 (± 4.430)	0.696

^a^Moderate intensity PA: the effort makes you warmer and your heart rate and breathing will be faster than normal. You may also sweat a little but will still be able to carry on a conversation

^b^Vigorous intensity PA: the effort makes your heart beat much faster and you have to breathe deeper and faster than normal. You will probably sweat.

#### Evidence collection

In total 40 students commented on their experience in the ‘MED-WELL’ programme using the free text boxes in the post-programme questionnaire.

#### Managing and analysing the empirical evidence

The five stages of the Framework Process were followed in the examination of the qualitative data which included familiarization, thematic framework identification, indexing, charting, mapping, and interpretation [27]. Coding was partially conducted with another researcher from a different professional background for inter-coder reliability [28]. Microsoft Excel was used to organize and code the data to facilitate the analysis and comparison of relationships between the coded ideas [29]. To heighten reflexivity three members of the research team (a nurse, a general practitioner and a medical student) reviewed the coded data and contributed to the thematic analysis [30].

#### Adopting an approach

The grounded theory approach was taken as it involves collection and analysis of the qualitative data. It outlines that the analysis and development of theories happens after the data has been collected [31].

#### Distillation of themes

The qualitative data was analysed to provide a general understanding of the data as whole. The data was then further explored to identify relationships between the main findings.

#### End point

The relationship between the key findings in the qualitative data and the feasibility of the ‘MED-WELL’ programme was explored.

### Potential to deliver the intervention

The potential for medical schools to deliver the intervention was also examined. To demonstrate the resource commitment necessary for the ‘MED-WELL’ programme the initial work, organization, and administrative support was documented and reported, which was done with the intention to support future exercise interventions in medical schools.

## Results

### Baseline characteristics of participants

Of all the participants 56 (78%) were female, compared to the overall study population which had 173 (61%) females. The mean age of all the participants was 25 years, with a range of 21 to 41 years; nearly all (*n* = 68, 94%) were non-smokers. Of note, 42 participants (58%) stated that their level of exercise had reduced since starting medical school yet 67 participants (93%) indicated having easy access to exercise resources. Close to half of the participants (*n* = 33, 46%) indicated having prior knowledge about the use of exercise as medicine for patients with chronic illness (Table 2).

### Feasibility

#### Recruitment and retention

**Fig. 2:**
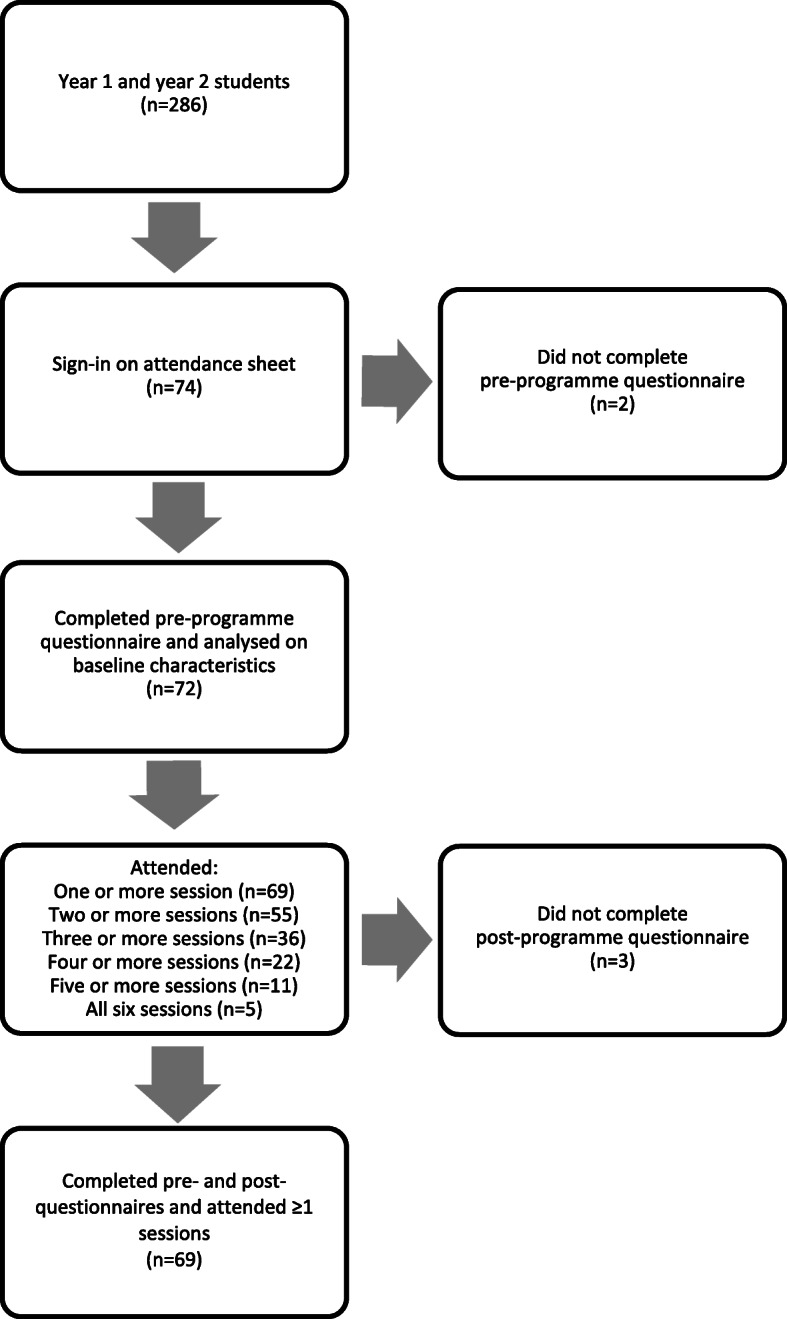
Participants in the 'MED-WELL' programme

At the time of the study there were a total of 286 graduate entry medical students enrolled in years one and two; of these, 74 graduate entry medical students (26%) took part in the study. Seventy-two (97%) completed the pre-programme questionnaire. Sixty-nine (93%) also completed the post-programme questionnaire and attended one or more of the ‘MED-WELL’ sessions. Data was analysed on these 69 participants (Fig. 2).

#### Assessment of outcome measures

Table 3 summarises efficacy outcome measures. Significant improvements were seen in self-reported PA during a typical week (*p* = 0.021); significant improvements were also seen in the WHO-5 Well-Being Index (*p* = 0.015), sleep patterns (*p* < 0.001) and loneliness scale (*p* = 0.003). Point estimates showed an improvement in EQ-5D-5 L-VAS, concentration, social support and participants’ perception of the importance of exercise as a treatment modality for common chronic conditions but these did not reach statistical significance.

#### Acceptability

Overall, 57 participants (84%) indicated ‘yes’ when asked if they would like to see the ‘MED-WELL’ programme held every year. 11 participants (16%) responded ‘yes with alterations’, zero participants indicated no and one participant left the question blank. 40 participants (58%) elaborated on their evaluation of the ‘MED-WELL’ programme via a free text box. When asked to elaborate on their evaluation most of the qualitative responses related to positive feedback regarding the ‘MED-WELL’ programme overall.

#### Qualitative analysis of free text

Three major themes that emerged from the qualitative data analysis were: activity preferences, curricular space, and benefits beyond fitness. These major themes were divided into a series of subthemes described below. All major themes and subthemes are summarized in Table 4.

**Table 4:** Summary of qualitative data in major themes and subthemes

Major theme	Subtheme
Activity preference	PA difficulty
	Instructor preference
Curricular space	Timing of exercise intervention
	Integration into curriculum
Benefits beyond fitness	Mental and social benefits
	Motivation to move

### Theme 1 - activity preferences

Activity preferences describe a theme of concepts that emerged from feedback pertaining to the type of PA or class offered at each weekly session. It contains the following subthemes: PA difficulty and instructor preference. These subthemes outlined how the variability of each weekly PA session affected the experience of the participants.

#### PA difficulty.

Table 1 outlines the exercise curriculum that was associated with each week of the ‘MED-WELL’ programme. Participants recognized the varying levels of intensity of each type of PA. It was apparent that some participants favoured certain types of PA within the curriculum based on their difficulty and asked for more of that type to be integrated into the ‘MED-WELL’ programme. The types of PA favoured ranged from low to high intensity which ultimately demonstrated the need for variety and modifications to different skill levels during the PA sessions.

*‘Less exercises that focus on breathing only and more fun, moderate activity’ [Participant 33].*

#### Instructor preference.

Participants shared that part of their experience in the ‘MED-WELL’ programme was reflected by the instructors that led the exercise curriculum each week. A clear perception emerged that participants had more positive experiences when instructors were encouraging to participants throughout the class. Participants with previous exposure to a certain type of PA also suggesting integrating different instructors into the ‘MED-WELL’ programme.

*‘Maybe use a different instructor. I’ve been to other pilates classes and it [MED-WELL] did not compare’ [Participant 43].*

### Theme 2 - curricular space

The second major theme was curricular space which described suggestions in how best to incorporate the ‘MED-WELL’ programme to suit the already-busy schedule of medical student. The subthemes that encompassed these suggestions were: timing of the exercise intervention and integration into the medical school curricula.

#### Timing of the exercise intervention.

The ‘MED-WELL’ programme was held on Wednesday mornings and although this time was preferred by some participants, others found it difficult to commit to that specific time. A few of the participants simply preferred to exercise in the evenings as opposed to mornings, while other participants requested the sessions be earlier in the morning. However there was an overall appreciation amongst participants for having the programme run at the same time every week as that made it easier to plan for in their own schedules.

*‘I found it difficult to commit to the same morning each week. So possibly have an evening option or choice of mornings’ [Participant 31].*

#### Integration into the medical school curricula.

Participants acknowledged that there was no part of the current curricula that offered PA or education on exercise as medicine and many of the participants envisioned the ‘MED-WELL’ programme as a solution to that gap. There was a clear concern towards making a PA-based programme mandatory as it may not suit the PA needs of every medical student. This related well to the major theme of activity preferences and how different PA level modifications could support a larger group of participants.

*‘I would love to see the ‘Med-Well’ programme take the place of our current Human Doctor module / be mixed with the module to show med students how to care for themselves with exercise’ [Participant 20].*

### Theme 3 - benefits beyond fitness

While the first two themes revolved around feedback towards the ‘MED-WELL’ programme and its overall implications, the third major theme of benefits beyond fitness describes what participants gained from the ‘MED-WELL’ programme themselves. Many of these benefits went beyond the opportunity to engage and learn about PA to include how it affected their mentality and actions. The subthemes here included mental and social benefits, and motivation to move.

#### Mental and social benefits.

Many of the participants expressed their satisfaction towards the positive effect the ‘MED-WELL’ programme had on their mental health and general wellbeing. Specifically participants mentioned lower stress levels and higher energy levels throughout the day after taking part in a weekly ‘MED-WELL’ session. Some participants also attributed these positive effects to the idea that the ‘MED-WELL’ programme was an opportunity to socialize with other medical students. Engaging with peers outside of a normal classroom setting was refreshing and contributed to an improved overall well-being.

*‘I think that exercise is extremely beneficial for everyone’s mental and physical health. I always feel less stressed and ready for the day after exercise’ [Participant 64].*

#### Motivation to move.

The ‘MED-WELL’ programme highlighted the importance of PA and helped motivate some participants to become more physically active. The idea that the ‘MED-WELL’ programme was not just informing on the benefits of exercise but encouraging participants to engage in PA was important to many participants. The subtheme of motivation to move encompasses the motivation to both attend the ‘MED-WELL’ sessions and engage in PA outside of the sessions.

*‘Although I did not get the chance to attend many this year, I think it’s a great activity and highlighted and reminded us of the importance of exercise’ [Participant 66].*

### Potential for medical schools to deliver the intervention

Delivery of the intervention required initial work including the composition of a nominal business plan, administrative support with organization and communication, and general follow-up. This information was formatted into a simple business plan to support the structure and organization of the intervention.

Initial work included acquiring ethical approval by applying to the local ethics committee for approval to proceed. Permission from the graduate entry medical school, UL to invite the medical students was also a prerequisite. Delivery of the intervention also involved securing the necessary funding (1500 euro over 6 weeks) and confirming insurance coverage for participants. This was achieved by facilitating the intervention via UL’s registered student-sports clubs.

Administrative support was provided ‘gratis’ by the medical school and was initially used to determine an appropriate venue and time for the intervention, which was done through consultation with the student body and faculty. Administrative support was then engaged to communicate with the student body before, during, and after the ‘MED-WELL’ programme. This communication included promotion of the programme, emailed and posted reminders of each weekly session, sharing the relevant slides through an online platform, collecting feedback from participants, and ensuring pre- and post-questionnaires were filled out. Generally, administrative support was extensively used to liaison with all parties involved (such as the educators, PA tutors, venue managers, student body, and faculty), ensure all necessary documents were present at each session (such as sign-in sheets, questionnaires, and informed consent forms), and ensure each weekly session and the intervention in its entirety was organized.

## Discussion

### Summary of main findings

This study has shown it is feasible to deliver the ‘MED-WELL’ programme as part of a medical school curriculum. Although the recruitment rate of participants was low at 26% the retention rate was high at 93%. The overall health and well-being of graduate entry medical students was positively affected by this exercise intervention with significant improvements demonstrated in self-reported PA during a typical week, WHO-5 Well-Being Index, sleep patterns, and 3-item loneliness scale.

There was no significant difference in participants’ perception of the importance of exercise as a treatment modality for common chronic conditions. However this may reflect a selection bias as participants had high levels of perception of importance at baseline, and participation was voluntary and not linked to assessment of the students in any way.

Although there was no significant difference in how important participants perceived exercise as a treatment modality for chronic conditions, the mean of results in the pre- and post-questionnaires was 3.65/4 and 3.81/4 respectively. These results indicate that even before the ‘MED-WELL’ programme the majority of participants perceived the importance of exercise as a treatment modality as ‘important’ or ‘very important’. This complements the result where almost half of participants acknowledged the use of exercise as medicine for patients with chronic illnesses before engaging in the ‘MED-WELL’ programme.

The qualitative analysis also demonstrated an acceptability of the ‘MED-WELL’ programme to students. Every participant indicated they would like to see the programme held annually, either with or without programme alterations.

### Comparison to existing literature

The significant difference in the WHO-5 Well-Being measurement demonstrates a positive change in mental well-being after the ‘MED-WELL’ programme consistent with other research relating positive mental health change to exercise [9, 11, 32]. The EQ-5D-5 L-VAS results also point to an improvement in overall health however the results fall just short of reaching statistical significance.

There is a wide variety of health promoting programmes in medical schools such as mindfulness-stress reduction, lifestyle programs, self-hypnosis, and yoga interventions; however a major limitation to the application of these programs is a lack of consistency and standardization [15, 33] which are elements addressed by the ‘MED-WELL’ programme.

An important factor in the ‘MED-WELL’ programme is the six-week timeline that it took place over. Exercise-based interventions in other institutions have various timelines from a few weeks [10] to months [15]. This suggests the need for a longitudinal study over a greater time period that would better represent the long-term behavioural changes that result from exercise interventions in medical schools.

The ‘MED-WELL’ programme involved only year one and year two medical students in part because year one of medical school is often a significant academic adjustment that is associated with a decrease in exercise throughout the year [34]. In the graduate entry medical school, UL year one and year two students similarly incorporate pre-clinical training in the form of PBL. Year three and year four students were not invited to participate in the study as these students are on clinical placements outside of the graduate entry medical school, UL.

Initial recognition regarding the importance of promoting exercise medicine in medical schools is not a new concept, with some UK institutions acknowledging that education should put a greater emphasis on exercise medicine more than 30 years ago [35]. There are many initiatives based in medical schools which encourage future healthcare providers to prescribe more exercise for patients due to its evidence-based role in improving health outcomes. However the focus of most exercise curricula in medical schools has been in the context of teaching modules that relate exercise medicine to chronic disease [36, 37] and not necessarily incorporating an educational aspect with the experiential aspect of the PA itself, as is seen with the ‘MED-WELL’ programme.

### Strengths and limitations

The strengths of this study are the clearly described procedures, the high assessment completion rate (93%), and the validated outcome measures used. However, this study also had a number of limitations. It was a small feasibility study conducted in a single medical school without a control group. There was also a low recruitment rate (24%) which may coincide with the fact that participation in the study was voluntary and not linked to assessment of the students in any way. The low recruitment rate may also indicate the process of recruiting students could be improved, or that the scheduled time for the ‘MED-WELL’ programme did not appeal to students. It is possible that the addition of the ‘MED-WELL’ programme to the medical school curriculum may increase participation rates.

### Implications for policy and research

Medical students have been shown to place poor prioritization on personal health, health promoting activities, and coping mechanisms [38]. By incorporating an exercise intervention into the medical curriculum medical students are provided with an accessible, consistent option for PA. Participant feedback suggests that by making the ‘MED-WELL’ programme a voluntary programme it is more appealing. This aligns with other studies that found optional participation in health promotion activities resulted in higher satisfaction rates and more positive engagement among students when compared to mandatory participation [39]. Our findings also indicated the majority of participants perceived exercise as important for their own personal health and as a treatment modality for chronic conditions, however that does not directly correlate with knowledge of current PA guidelines and recommendations of exercise to healthy adults [40]. An important implication of this intervention is the benefit that it will extend to patients of these medical students in the future as research demonstrates that doctors that are physically active are more likely to prescribe exercise for their patients [41].

We also observed the variety of the type of PA offered at each weekly session as important to the participants. Offering more vigorous activity (such as high intensity interval training) as well as more passive activity (such as sports yoga) gave participants the opportunity to try different activities while still being able to modify each activity to suit their fitness level.

## Conclusions

This study has shown it is feasible to deliver an exercise intervention - the ‘MED-WELL’ programme - to educate and promote health and well-being among medical students. Qualitative responses about the ‘MED-WELL’ programme were significantly positive and all in favour of seeing the intervention continue annually. Well-designed randomised controlled trials are needed to measure outcomes, durability of effect, and cost effectiveness.

## Data Availability

The datasets used and analysed during this study are available from the corresponding author upon reasonable request.

## Abbreviations

PA: Physical Activity
UL: University of Limerick
PBL: Problem-Based Learning
